# Carbon Nanotube Modified Microelectrode Array for Neural Interface

**DOI:** 10.3389/fbioe.2020.582713

**Published:** 2021-01-13

**Authors:** Mohaddeseh Vafaiee, Raheleh Mohammadpour, Manouchehr Vossoughi, Elham Asadian, Mahyar Janahmadi, Pezhman Sasanpour

**Affiliations:** ^1^Institute for Nanoscience and Nanotechnology, Sharif University of Technology, Tehran, Iran; ^2^Department of Chemical and Petroleum Engineering, Sharif University of Technology, Tehran, Iran; ^3^Department of Medical Physics and Biomedical Engineering, School of Medicine, Shahid Beheshti University of Medical Sciences, Tehran, Iran; ^4^Neuroscience Research Center and Department of Physiology, School of Medicine, Shahid Beheshti University of Medical Sciences, Tehran, Iran

**Keywords:** carbon nanotubes, neural interface, multi-electrode arrays, electrochemical impedance, neural recording

## Abstract

Carbon nanotubes (CNTs) coatings have been shown over the past few years as a promising material for neural interface applications. In particular, in the field of nerve implants, CNTs have fundamental advantages due to their unique mechanical and electrical properties. In this study, carbon nanotubes multi-electrode arrays (CNT-modified-Au MEAs) were fabricated based on gold multi-electrode arrays (Au-MEAs). The electrochemical impedance spectra of CNT-modified-Au MEA and Au-MEA were compared employing equivalent circuit models. In comparison with Au-MEA (17 Ω), CNT-modified-Au MEA (8 Ω) lowered the overall impedance of the electrode at 1 kHz by 50%. The results showed that CNT-modified-Au MEAs have good properties such as low impedance, high stability and durability, as well as scratch resistance, which makes them appropriate for long-term application in neural interfaces.

## Introduction

Over the years, Multi-electrode arrays (MEAs) technologies have been widely used in a variety of neuroscience and cardiology applications to study *in vitro* as well as *in vivo* bioelectric signals of electrogenic cells (Kotov et al., 2009; Kim et al., 2014; Kuzum et al., 2014). MEAs are composed of a cell culture platform with electrical interfaces for recording and stimulating electrogenic cells (Nam, 2012; Zhang et al., 2012; Vafaiee et al., 2018). These platforms are used for cardiac or neural cells and tissue slices studies (Qing et al., 2010; Fattahi et al., 2014), prostheses and rehabilitation (Kipke et al., 2008; Jorfi et al., 2016), deep brain stimulation (DBS) (Butson and McIntyre, 2005; Parastarfeizabadi and Kouzani, 2017), cardiac pacemakers (Nick et al., 2012), electrocorticography (ECoG) (Buzsáki et al., 2012), retinal (Grumet et al., 2000; Ahuja et al., 2008) and cochlear implants (Dhanasingh and Jolly, 2017; Senn et al., 2017), or for Brain-computer interfaces in general (Wise et al., 2008; Rothschild, 2010).

Thin films of transition metals such as gold (Dimaki et al., 2014; Vafaiee et al., 2019), platinum (Brummer and Turner, 1977; Desai et al., 2010), and iridium (Eick et al., 2009; Brunton, 2015) are usually used for the construction of microelectrode arrays due to their high conductivity and biocompatibility (Blau et al., 2011). Given the importance of electrode size in the recorded signals, achieving less impedance in electrode construction is the crucial factor. A high-impedance electrode makes unreliable measurements and significantly leads to considerable thermal noise (Heim et al., 2012). Moreover, a high impedance electrode cannot provide sufficient charge to the cells or tissues before it encounters irreversible electrochemical reactions that may damage the cells or the electrode itself (Cogan, 2008). Therefore, to ensure a high signal-to-noise ratio (SNR) and effective cellular stimulation, it is necessary to choose a material as an electrode that has high conductivity and fast electron transfer kinetics. One of the most widely used methods to reduce impedance and improve SNR is surface engineering to provide micro and nanoscale interfaces (Nam, 2012; Kim et al., 2014). In this regard, a variety of materials and morphologies have been utilized. The applicability of emerging technology especially in the field of neural engineering and neurobiology have been remarkably increased with the recent advances in the field of nanotechnology and micromechanics. Various kinds of nanomaterials such as conducting polymers (CPs), graphene and CNTs are considered as attractive candidates to study the nervous system and have been successfully applied for signal recording, nerve cell stimulation, and nerve cell growth (Kotov et al., 2009; Abidian et al., 2010; Nam, 2012; Sasanpour et al., 2014). The application of these new materials has created different advantages and disadvantages. For example, using conductive polymers, along with improving the adhesion of the electrode, complicates and prolongs the manufacturing process of the electrode, and on the other hand, requires a series of steps to remove the residues of polymerization solutions.

Carbon nanotubes are hollow cylinders of graphite sheets with the diameter of the order of nanometers and include single-walled (SWCNTs) and multi-walled (MWCNTs) carbon nanotubes (Soldano et al., 2013). These 1D materials possess excellent properties such as high surface area, excellent electrical conductivity, high mechanical strength, biocompatibility (Warheit et al., 2004; Firme and Bandaru, 2010) as well as the ability to provide excellent support for nerve cell adhesion (Malarkey and Parpura, 2007; Voge and Stegemann, 2011) which make them an interesting material as the interface between cells and electrodes. Recent studies have confirmed the enormous potential of CNT as a biocompatible platform to which neurons can easily adhere (Hu et al., 2005; Malarkey and Parpura, 2007; Ghasemi-mobarakeh et al., 2011). In fact, carbon nanotubes can be tightly wrapped by cells, which reduces the distance between the membrane and the electrode and hence, increases the seal resistance (Hu et al., 2005; Jeong et al., 2016). These properties are related to their surface characteristics such as roughness, polarity, charge and their chemistry (Hu et al., 2004; Malarkey et al., 2009). High surface area can lead to a significant increase in the charge injection capacity and a decrease in the interfacial impedance (Keefer et al., 2008). In summary, carbon nanotubes have a unique combination of properties, including (1) electrical conductivity, (2) good electron transfer (Nugent et al., 2001), (3) high surface-to-volume ratio, (4) ease of biomolecular modification (Shim et al., 2002; Bekyarova et al., 2005; Lu et al., 2009; Bottini et al., 2011), (5) optimal mechanical properties (6) surface roughness and three-dimensional structure (3D) that strengthens cellular interfaces. Owing to these desirable properties, CNTs are intriguing candidates for neural interface electrodes (Fung et al., 2010; Jeong et al., 2016).

In the present study, surface modification of gold multielectrode arrays by carbon nanotubes has been adopted as the main strategy to reduce the impedance of the prepared platform. To this end, high scratch strength gold electrodes (Au MEAs) were made in the first step using the usual three-layer configuration followed by drop-casting of multi-walled carbon nanotubes to the surface of the electrode sites (CNT-modified-Au MEA). Various methods have been used to incorporate carbon nanotubes to the electrode structure, including the direct growth of nanotubes on the electrode, which requires the presence of catalysts (Nick et al., 2012; Jeong et al., 2015, 2016; Morassutto et al., 2016), and embedding nanotubes inside the electrode structure using additional materials to create the necessary adhesion (Gerwig et al., 2012; Castagnola et al., 2014; Kozai et al., 2015; Carli et al., 2018). In the present study, we have employed a facile method to embed the nanotubes in the structure of the electrode without the need for any additional adhesive, catalyst or further steps in the construction process of the electrode. To this end, the surface of gold substrate was roughened by the aid of a surface modification process on the base electrode which made the nanotubes to be well attached to the substrate and as a result, no detachment was observed during the operations in the presence of biological solutions. Hence, the proposed method not only reduces the steps and cost of construction, but also will improve the biocompatibility of the electrode, since there would be no additional or residual material on the surface of array during the work process. In this regard, the adhesion of CNT to the electrode and preventing the detachment of nanotubes from the substrate in the process in the solution has been improved effectively. Optimal adhesion between carbon nanotubes and electrode surfaces was achieved due to the special properties of gold electrodes with high scratch strength. The identification of electrodes by electrochemical methods is an important step in predicting *in vivo* performance of the electrodes (Stacey et al., 2016). The extracellular recording and stimulation ability of the CNT-modified electrodes were investigated by means of electrochemical impedance spectroscopy (EIS) to assess their *in vivo* performance. In addition, the equivalent electrical circuits were extracted to achieve a more accurate and profound understanding of the electrodes’ behavior.

## Materials and Methods

### Device Fabrication

**FIGURE 1:**
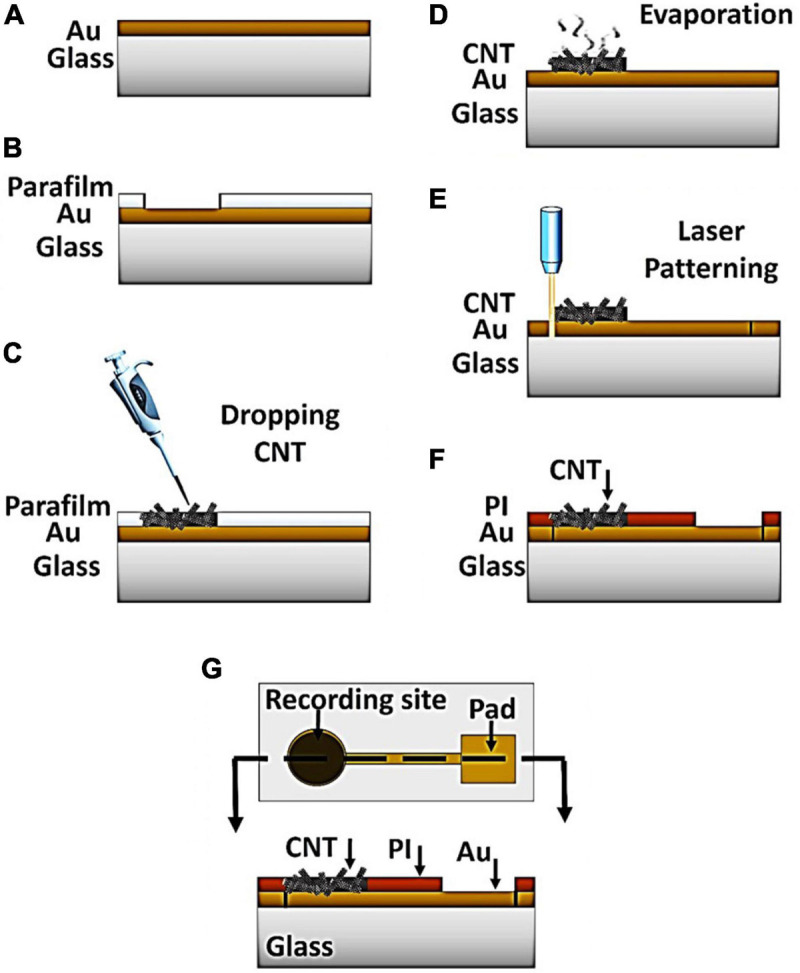
Process of the CNT-modified-Au device fabrication. **(A)** Gold sputtering on the glass substrate. **(B)** Placement of parafilm layer on the sample to provide a fixed area **(C)** Drop casting of CNT suspension into the exposed area **(D)** Solvent evaporation at room temperature for 30 min **(E)** Patterning the electrode by laser **(F)** Insulation of the electrode surface by PI polymer **(G)** top and side view of the device.

The CNT-modified-Au MEAs were fabricated on a glass substrate in a three-step procedure including sputtering the gold layer followed by drop-casting of CNTs suspension and final patterning by laser. The fabrication steps are schematically illustrated in Figure 1. Briefly, the glass substrate was first subjected to the surface modification to achieve adequate adhesion of the gold layer to the substrates’ surface without the need of adhesive layers (Vafaiee et al., 2019). The glass substrate was chosen in order to provide the necessary mechanical strength during the handling. Moreover, glass as an insulating substrate provides the required transparency, biocompatibility, as well as temperature resistance. A thin layer of gold with a thickness of 100 nm was then deposited on the modified glass substrate using the sputtering method (Desk Sputter Coater-DST3-A, Nanostructured Coating Co., Iran) (Figure 1A). Gold was chosen as the conductive layer due to its biocompatibility, high electrical conductivity and inertness in biological solutions, which are critical factors for more accurate recording and long-term durability of the electrode (Gross, 1979). Carbon nanotubes were used not only to increase the electrical conductivity of the electrode, but also to improve the adhesion of the cell to the electrode surface. Multi-walled carbon nanotubes with hydroxyl functional groups were purchased from Neutrino Company. A highly dispersed suspension of carbon nanotubes (1 mg/mL) in deionized water (DI) was prepared by the sonication for 30 min in an ultrasonic device. In order to keep the electrode surface modification conditions constant, a piece of parafilm with a 1 × 1 cm^2^ window was placed on the electrode surface (Figures 1B,C). Then, 20 μL of CNT-OH was drop-casted in the exposed area and the electrode was let to be dried in the ambient temperature for 30 min (Figure 1D), after which the nanotubes adhered to the electrode surface. The electrode was consequently washed with deionized water to remove the unattached nanotubes. The electrode patterns were made by irradiation of the samples with the YW-300B laser (power 20 W, wavelength 1.06 μm, frequency 50/60 Hz) of the PERSONAGE INT. CO. LTD (Figure 1E). The thickness and depth of the created lines is dependent on the speed and power of the laser radiation. Due to the increased thickness at the carbon nanotube suspension drop point, the applied laser power and speed have been adjusted so that the electrical connection of the created paths and pads disconnected from each other. The electrode was finally insulated using a thin Polyimide (PI) layer (Figure 1F), which is biocompatible (Myllymaa et al., 2009; Xue et al., 2018). Furthermore, the PI layer has a high thermal resistance which makes the final electrode compatible with the necessary conditions for sterilization in an autoclave (high temperature and pressure). The polymeric coverage isolated the surface of the electrode from biological solutions except for the recording sites. A cell chamber that could maintain the cultured cells and the electrolyte solution was fabricated by a glass cylinder that was attached to the electrode surface using biocompatible polydimethylsiloxane (PDMS). The sealing efficiency was checked and confirmed using DI water. The electrode’s constituents were selected in such a way that 70% alcohol and UV light as well as autoclave can be used to sterilize the electrode if necessary.

### Device Characterization

#### Microscopic Analysis

The morphology of the electrode surface as well as the nanostructures’ composition were investigated by using a field emission scanning electron microscopy (FESEM, TESCAN MIRA3, Czechia). An optical microscope equipped with a digital camera was utilized to observe the dimensions and position of the electrodes relative to each other (Dino-Lite Digital Microscope).

#### Electrochemical Characterization

Electrochemical impedance spectroscopy (EIS) is a non-destructive electrochemical method commonly used to characterize the electrode’s surface properties as a measurement of the system’s response to alternating current (Lust et al., 2003; Abidian and Martin, 2008; Kusko et al., 2012; Vafaiee et al., 2019). The most important feature of the array electrodes for the signal recording application is the electrode impedance, which is effective in recording signals with greater accuracy and lower noise levels. This is due to the fact that the thermal noise associated with the resistance component of the impedance is a significant source of noise in neural recordings. The lower the impedance of an electrode with a specified geometric area, the better the signal performance. In fact, in electrochemical impedance spectroscopy (EIS), the system’s response to an alternating current is investigated and the behavior of the electrode at different frequencies in the biological solution is examined. Finally, as a criterion for comparison, the value of the electrode impedance at the frequency of 1 kHz which is the standard frequency in the signal recording range of nerve cells, is expressed. EIS electrochemical analysis was performed using an Autolab electrochemical workstation (PGSTAT 302N) in the presence of phosphate-buffered saline (PBS) as the electrolyte solution to mimic the biological environment. Electrochemical measurements were conducted in a standard three electrode cell configuration, in which the fabricated electrode sites served as working electrodes with a Pt wire and an Ag/AgCl electrode as counter and reference electrodes, respectively. Prior to the EIS measurements, the electrodes were electrochemically cleaned by applying cyclic voltammetry in the range of −1 to 1 V vs. Ag/AgCl (at the 50 mV/s scanning rate). Afterward, EIS plots were recorded in the frequency range of 1–10 MHz with a potential amplitude of 10 mV rms under an open circuit DC potential.

In the present study, not only the performance of the electrode in the signal recording was investigated by the aid of EIS technique, but also the electrical modeling of the electrode behavior was done which provides valuable information on how the electrode works. For this purpose, the resulted Nyquist plots were fitted using ZView software.

#### Stability and Durability

For a modified electrode to be practical, mechanical stability and long-term durability are two critical factors needed to be carefully considered. In this respect, the stability of various electrodes was studied under experimental conditions, i.e., in the presence of sterilizing and biological solutions such as cell culture media or ACSF solution. For this purpose, the condition of the electrode surface was checked, once before the exposure of the electrodes to ACSF and ethanol 70% and once after that for 30 min. Moreover, in order to evaluate the stability and durability of the electrode, electrochemical impedance spectroscopy (EIS) technique was used as an accurate method which shows high sensitivity to the surface roughness and porosity of the electrode (Randviir and Banks, 2013). The electrode was placed in the cell culture medium (RPMI) and ACSF solution and the impedance of the samples was measured after 0, 1, 5, and 10 days and each measurement was repeated 5 times.

#### Signal Recording

The performance of the electrodes was checked by signal recording suing the hippocampal slices of the Wistar male rat of the 35- to 45-day-old. Mice were deeply anesthetized with ether and then, the animal was decapitated and the brain was quickly removed and placed in the cold carbogenated sucrose-based ACSF containing (in mM): 206 sucrose, 2.8 KCl, 1 CaCl_2_, 1 MgCl_2_, 2 MgSO_4_, 1.25 NaH_2_PO_4_, 26 NaHCO_3_, 10 D-glucose (pH 7.3–7.4; 295 mOsm). Transverse hippocampal slices of 300–400 μm thick were prepared using a vibrating slicer (752 HA, Campden Instruments Ltd., United Kingdom) and transferred to an incubation chamber containing modified ACSF composed of (in mM) 125 NaCl, 2.5 KCl, 1.5 CaCl_2_, 1.25 NaH_2_PO_4_, 25 NaHCO_3_, 10 D-glucose, saturated with 95% O_2_ and 5% CO_2_ (pH 7.4, 295 mOsm) for at least 45 min at 32–35°C. Then, slices were kept at room temperature (22–24°C) in a holding chamber and continuously perfused with ACSF oxygenated with 95% O_2_ and 5% CO_2_ for at least 1 h before recording. Then, slices were transferred to the CNT-modified-Au MEA for recording. All the animal experiment procedures were performed according to the guidelines of the ethics committee of Neuroscience Research Center and approved by the Institutional Ethics Committee at Shahid Beheshti University of Medical Sciences under permit number 25–12–88358.

## Results

**FIGURE 2:**
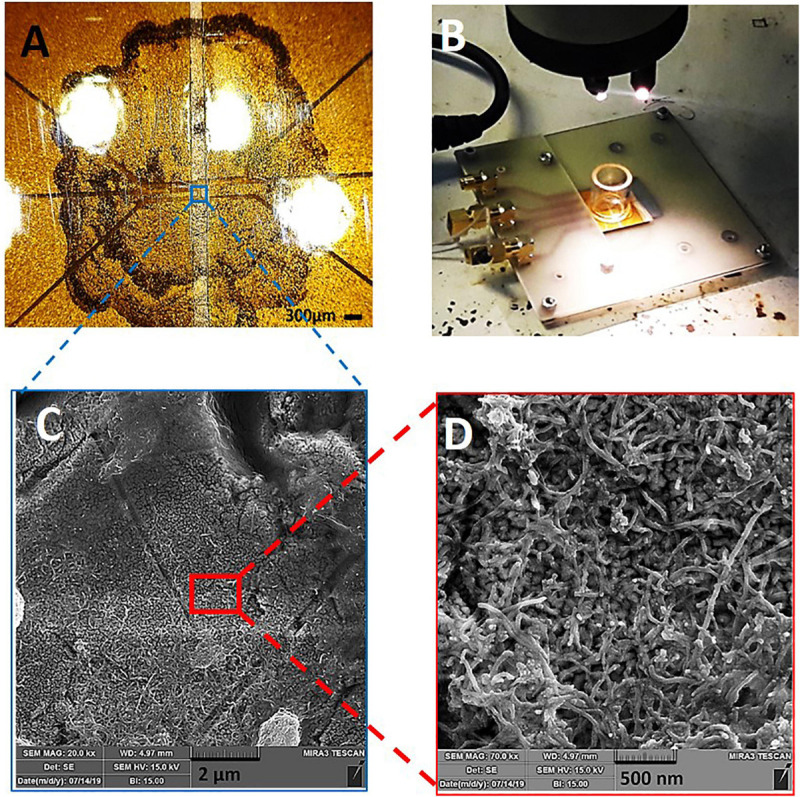
CNT-modified-Au device: **(A)** Optical image of the electrode surface, **(B)** Electrode board device with culture chamber and PCB connections, **(C,D)** SEM of the electrode surface containing CNTs with different magnifications.

Figure 2 presents the images of CNT-modified-Au electrodes. As shown in Figure 2A, the central part of the electrode contains a drop of carbon nanotubes that covers the electrode active area. Figure 2B depicts a typical device consisting of a glass substrate (4 × 2.5 cm^2^) on the surface of which five CNT electrodes and a glass chamber with a diameter of 2 cm for cell culture and electrochemical experiments, as well as PCB connections are integrated. Recording electrodes with a diameter of 100 micrometers are separated by 200 micrometers interval distance. The obtained SEM images from the electrode surface with different magnifications show a thin layer of CNTs which homogeneously covers the electrode surface (Figures 2C,D).

**FIGURE 3:**
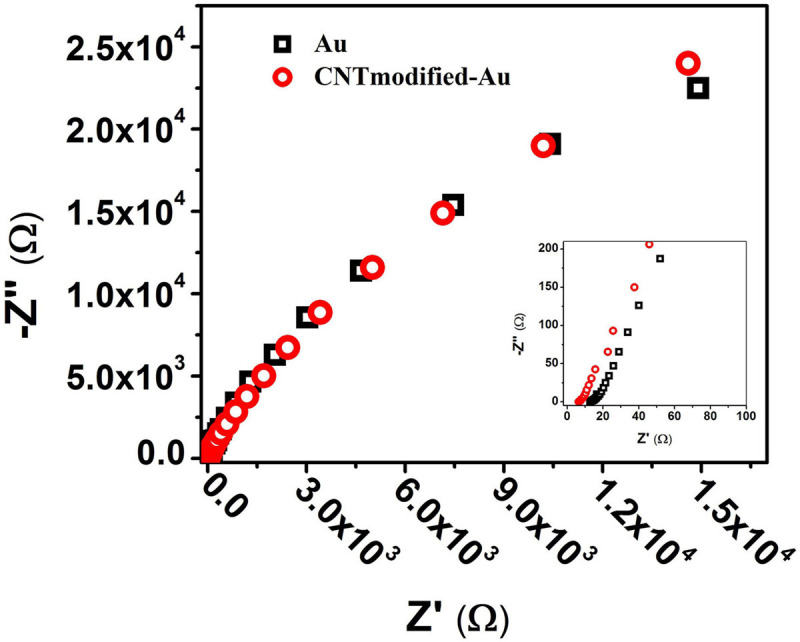
Nyquist diagram from the EIS test for Au (black) and CNT-modified-Au (red) electrodes. *Inset*: shows an enlarged view of the beginning of the chart.

The EIS results for Au and CNT-modified-Au electrodes are shown in Figure 3. As can be seen in the diagram, the general electrochemical behavior of the CNT-modified-Au electrode is similar to that of Au electrodes. The inset shows an enlarged view of the EIS plot in the high-frequency region. The semi-circle portion observed in the Nyquist plot at high-frequencies corresponds to the electron transfer limiting process and is a good measure of the charge transfer resistance (Rct). The results indicate that by modifying the Au electrode surface with CNTs, the electrical conductivity is increased and hence, the charge transfer resistance is reduced.

Previous electrophysiological measurements exhibited that the Nyquist diagram has a large semicircle due to the high amount of charge transfer resistance in the PBS solution used in the experiments to mimic the biological system environment (Wierzbicki, 2013). As shown in Figure 3, the Nyquist plot shows no semicircle since there is no active species in the PBS electrolyte solution which is consistent with previous results. In such cases, the Bode plots provide more information compared to that of Nyquist.

**FIGURE 4:**
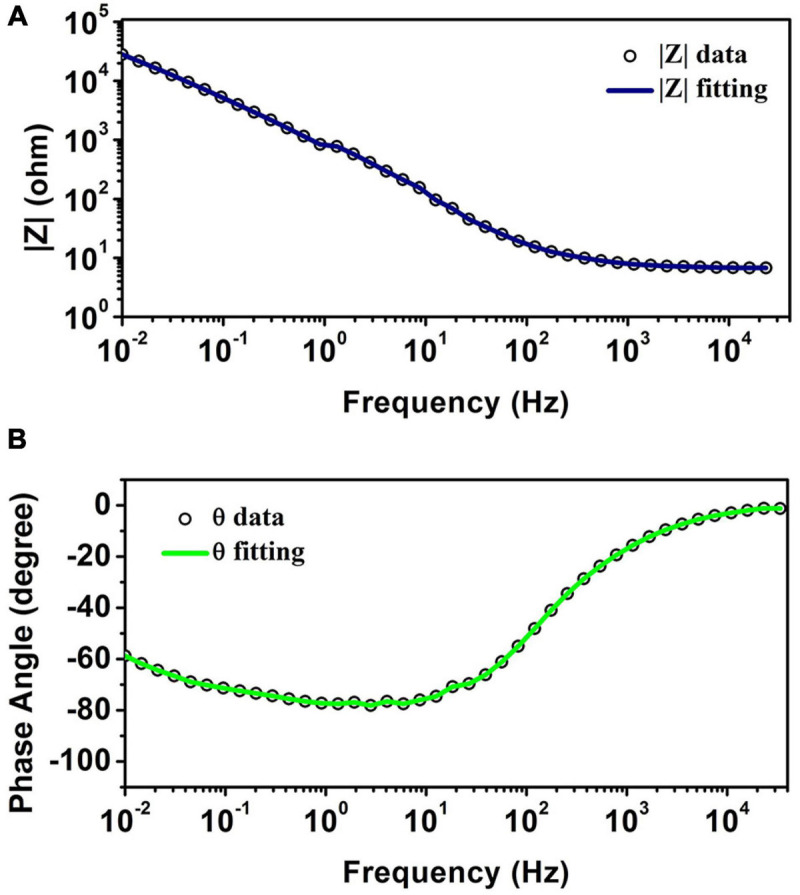
**(A)** The magnitude of impedance spectra. **(B)** The phase of impedance spectra obtained from EIS test for CNT-modified-Au electrodes.

Figure 4 illustrates the Bode plot of CNT-modified-Au electrodes according to which the electrode exhibits capacitive behavior. As a matter of fact, the phase angle closer to 90 degrees represents more capacitive behavior, while reduction of phase angle to zero indicates the resistive behavior (Kuzum et al., 2014). At high frequencies, the system’s behavior moves from capacitive toward resistive. In this region, the bulk resistance with zero phase is predominant. In the middle frequencies, the phase is in the range of −90°, which indicates the capacitive behavior of the electrode. The negative slope of the magnitude spectra also suggests the capacitive behavior of the electrode in this frequency range. The low frequency domain is usually dominated by charge transfer resistance and presents itself as a fixed magnitude where the phase is constant at zero degrees and as the results show, the capacitive effect is still in this range. Impedance | Z| at 1 kHz, which is the range of frequencies observed in neural signals, is a standard reference value for neural recording electrodes (Williams et al., 2007; Park et al., 2014). The impedance of the electrode at a frequency of 1 kHz is approximately 8 Ω.

**FIGURE 5:**
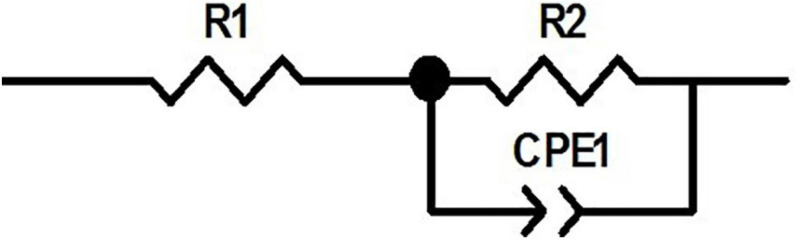
The equivalent circuit for the electrode.

According to the observed behavior of the electrode, the equivalent circuit shown in Figure 5 was considered for the system, which is a series-parallel circuit including charge transfer resistance (R2), electrical double-layer capacitor (CPE1) and the solution resistance (R1) (Wang et al., 2006; Jeong et al., 2016). Equation 1 shows the total impedance of the circuit accordingly.

(1) $$Z_{\mathrm{total}}=R_{s}+\frac{1}{\frac{1}{Z\,\left(C_{\mathrm{dl}}\right)}+\frac{1}{R_{\mathrm{ct}}}}$$

ZView software was used for data fitting with the proposed equivalent circuit. Table 1 shows the values corresponding to the elements of the equivalent circuit based on the results of data fitting. The charge transfer resistance value was obtained 76 kΩ, which is related to the system’s electrolyte solution (PBS), which lacks active species. CPE describes a non-ideal capacitor with the impedance Z_CPE_ = [q (jw) ^n^]^–1^, where q represents the capacitive behavior and n is the deviation from the ideal behavior. For *n* = 1, CPE is like an ideal capacitor (Bard et al., 1980; Yi et al., 2015; Vafaiee et al., 2018). The value of *n* = 0.847 obtained for the electrode (which is close to 1) denotes that the constant phase element is close to the behavior of an ideal capacitor.

**TABLE 1:** The values of the circuit elements corresponding to the equivalent circuit for the CNT-modified-Au electrode compared to the gold electrode.

	**R1 = Rs**	**R2 = Rct**	**CPE1-T**	**CPE1-P**
	
			**q**	**n**
CNT	6.82	76 × 10^3^	26.99 × 10^–5^	0.84
Au	12.65	59 × 10^3^	12.43 × 10^–5^	0.86

**FIGURE 6:**
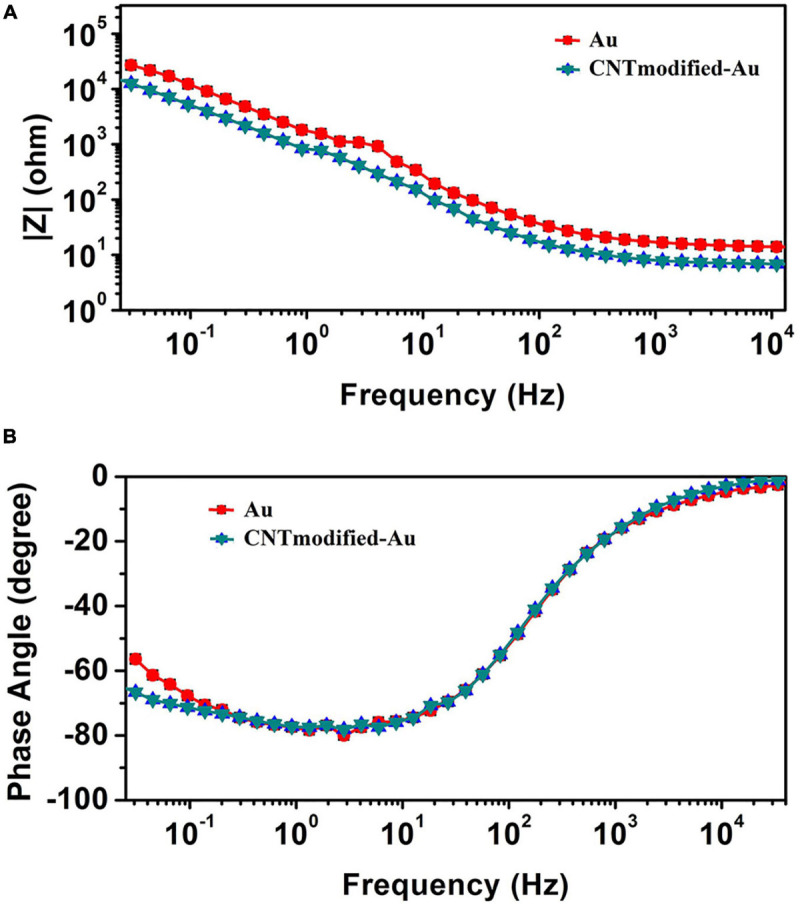
**(A)** The magnitude and **(B)** the phase of impedance spectra obtained from EIS test for CNT-modified-Au electrode (blue) and Au electrode (red).

Figure 6 shows the Bode plots of the CNT modified-Au electrode compared to the Au electrode. Since the value of the geometric area of the electrode affects the electrochemical results (Vafaiee et al., 2018), the same geometric area was selected in the test for two electrodes. Based on the amplitude charts depicted in Figure 6, the addition of CNTs was shown to reduce the impedance relative to the Au electrode, which can be attributed to the presence of CNTs that, as a conductive network, enhance the specific conductivity of the electrode (Gerwig et al., 2012). In fact, the Bode plot of the EIS data indicated that the CNT-modified-Au electrode had a significantly lower overall impedance than the Au electrode at all frequencies. Both curves show the same characteristic of cut-off frequency. As mentioned, the negative slope of the magnitude chart indicates the predominant capacitive effect of the circuit, which was observed at low frequencies for the present electrodes. In this region, a larger electric double layer capacitance would result in a lower magnitude diagram. As can be seen, the value of the electric double layer capacitance is smaller in the case of Au electrode and larger for the electrode containing CNTs. At the frequency of 1 kHz [the usual frequency observed in neural signals (Jeong et al., 2016)], the impedance of the gold electrode is approximately 17 Ω. In fact, the presence of carbon nanotubes reduced the amount of electrode impedance by 50%. The CNT-modified-Au electrode has a phase diagram similar to that of the Au electrode, which points out that the circuit is approximately the same for two electrodes. Therefore, for fitting the electrochemical results of the Au electrode, the same equivalent circuit was used, and the values of the obtained circuit elements are given in Table 1 compared to the CNT-modified-Au electrode. Based on the results shown in Table 1, the value of Rs for CNT-modified-Au electrodes is significantly lower than that of Au electrodes. Since the conductive substrate used for the CNT-modified-Au electrode is the same as the Au electrode, the conductivity of the electrode at the electrode active areas was increased with the addition of CNTs, and the amount of Rs resistance was decreased.

**FIGURE 7:**
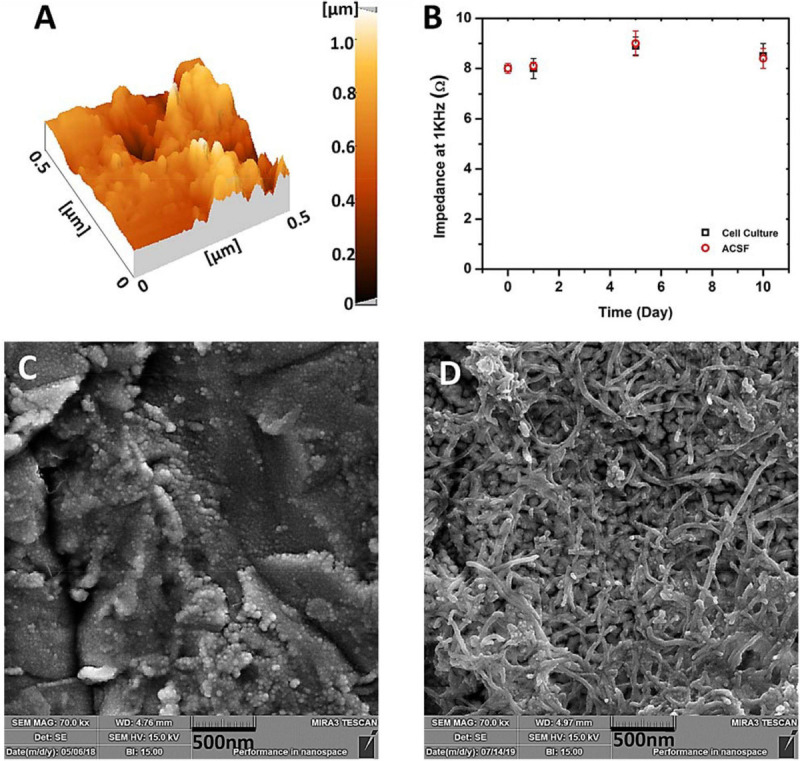
**(A)** AFM images of the Au electrode surface, **(B)** Measurement of the impedance of CNT modified-Au electrode at 1 kHz versus days kept in cell culture (RPMI) and ACSF solution (The error bars are for 5 replicate measurements), **(C)** SEM images of the Au electrode surface, and **(D)** SEM images of the CNT-modified-Au surface.

As mentioned earlier, to determine the stability and durability of the electrode during the sterilization, cell culture and recording processes the electrode was tested in the presence of the required solutions and under the working conditions. The electrodes were then checked and the results show no difference which demonstrates the good mechanical adherence of the CNT and gold layer to the surface of the glass substrate. The results revealed that the electrodes were not damaged during handling or sterilization, and the CNTs were not washed or detached from the surface while using alcohol or ACSF solutions which indicate the stability of the prepared electrodes. The obtained mechanical stability of the modified electrode is the result of high scratch-strength of gold layer as the base electrode and the good adhesion of CNT conductive film which turns the prepared electrode to a suitable platform for the next steps of recording due to its great durability and stability (Vafaiee et al., 2019). AFM image (DME Nanotechnologie GmbH, DS 95 Series) of the gold electrodes’ surface (Figure 7A) shows high roughness that provides a favorable platform for excellent adhesion of nanotubes. As can be seen in Figures 7A,C, the surface of the base gold electrode has random roughness. By tightening and entangling the carbon nanotubes between these rough structures, a strong adhesion was established as shown in Figure 7D. Figure 7B depicts the impedance of the CNT-modified-Au electrode which was measured at the frequency of 1 kHz. As shown in the plot, the measured impedance values of CNT-modified-Au electrodes did not change significantly even after 10 days of storage in the cell culture medium or ACSF, which indicates that the electrode has good stability and durability.

**FIGURE 8:**
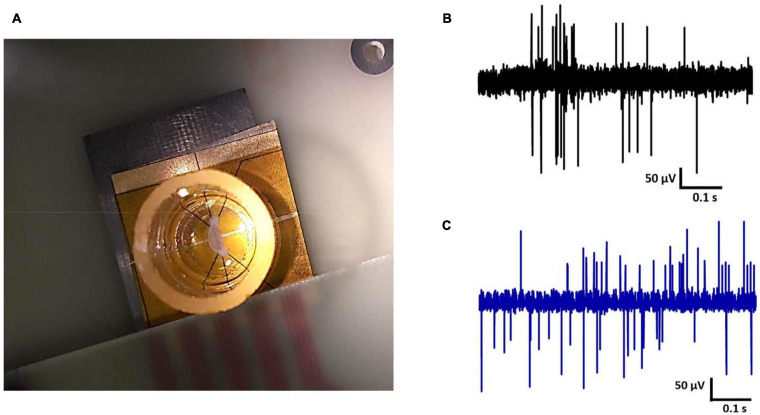
**(A)** Hippocampal tissue slice on the electrode, electrical activity of rat hippocampal neurons recorded by **(B)** Au MEA and **(C)** the CNT-modified-Au MEA.

Spontaneous electrical signals from rat hippocampal slices were measured on the Au and CNT-modified-Au electrodes using our lab made low noise measurement system. Signals were recorded with an acceptable range and signal-to-noise ratio. Figure 8 demonstrates a hippocampal tissue slice on the electrode (Figure 8A) and the recorded signals (Figures 8B,C). As can be seen in the Figure 8, the signal recorded by the CNT-modified-Au electrode has lower background noise comparing with the Au electrode. The prepared modified electrode in this way demonstrated good electrical properties during recording experiments and could register signals properly.

## Discussion

In this study, a new microelectrode array as neural interfaces has been fabricated. The electrode structure was characterized by various methods including microscopic imaging, electrochemical characterization, investigating the stability and durability of the electrode in the presence of biological solutions and signal recording from hippocampal tissue to evaluate its performance. The results showed that the electrode has suitable properties for recording neural signals.

The CNT-modified-Au arrays compared with Au arrays offered desirable features, including (1) low impedance, which leads to more electrical conductivity and the construction of smaller electrodes, (2) high stability and durability in the presence of biological solutions that lead to long-term use of the electrode. (3) Lower background noise levels in recorded signal from hippocampal tissue, which increases recording accuracy and improves the signal-to-noise ratio of the system.

Carbon nanotubes have been used to obtain the improved properties of gold electrodes. For this purpose, using surface changes in the structure of the base electrode, roughness was created on the surface of the gold substrate, which caused a good connection of the nanotubes to the substrate. The applied method for embedding the nanotubes in the electrode structure does not require any additional adhesive or catalyst and additional steps in the construction of the electrode. It was observed that during the recording process and in the presence of biological solutions, no separation of nanotubes from the surface occurred. In this regard, the properties of the electrodes didn’t change significantly. In addition to reducing the cost and steps of fabrication, it will improve the biocompatibility of the electrode as there will be no additional or residual materials from the process. The fabricated electrodes exhibit lower impedance compared to the base gold electrode and also lower noise during recording. The stability and durability of the fabricated electrodes are appropriate, which will allow long-term use of the electrodes. As can be clearly seen in the SEM images, the nanotubes not only increase the surface roughness and as a result, enhance the effective surface area of the electrode, but also they provide a suitable platform for the growth of nerve cells due to their stretched and strip structures. Recent studies have shown that surface topography is an important parameter that affects the anchoring properties and nerve branching (Ghasemi-Mobarakeh et al., 2008; Hoffman-kim et al., 2010; Roach et al., 2010). In general, the origin of the interaction of neurons and CNTs seems to be strongly influenced by surface roughness. Previous investigations suggested that the roughness of CNTs helps to anchor nerve cells (Sorkin et al., 2009). Since cells adhere to rough surfaces when exposed to the same chemistry (Fan et al., 2002). Nanotubes provide a large surface area by creating a porous structure, which can allow the ions to diffuse into the interior (Gerwig et al., 2012). The choice of substrate supportive material is very important for the successful deposition of CNTs. Herein, the surface roughness of the base gold electrode plays a critical role in improving the adhesion of carbon nanotubes to the surface (Vafaiee et al., 2019).

## Conclusion

Carbon nanotubes modified-Au MEA has been fabricated and its improved electrochemical properties in combination with the simple and cost-effective preparation method as well as stability and durability turn to a promising platform for neural applications have been studied. The results showed a larger capacitance and lower resistance than the base gold MEA, which reduces the impedance and thus diminishes the noise level of the final device. The prepared modified electrode revealed high mechanical stability even after exposure to the biological solutions for a long period without changes in the electrode surface properties and its impedance which indicates that the carbon nanotubes considerably adhered to the surface as a result of the roughness of the gold substrate. In addition, the similarity of CNT surfaces with the nanostructured properties of natural neural tissue makes CNTs a good interface for neural applications. The high electrical conductivity of CNTs allows also a direct electrical connection with neurons. These benefits of CNT-modified-Au electrodes could be useful in basic science research to understand the neural function as well as for the drug screening, neurotoxicity assessment, and cell signal transduction researches.

## Data Availability Statement

The raw data supporting the conclusion of this article will be made available by the authors, without undue reservation.

## Ethics Statement

The animal study was reviewed and approved by Ethics Committee of Neuroscience Research Center and approved by the Institutional Ethics Committee at Shahid Beheshti University of Medical Sciences under permit number 25–12–88358.

## Author Contributions

PS, RM, and MVo conceived the project and designed the research. MVa performed the experiments. RM, PS, EA, and MVa analyzed and interpreted the results. MJ, MVa, and PS performed the animal experiments and interpreted the results data. All authors read and edited the manuscript.

## Conflict of Interest

The authors declare that the research was conducted in the absence of any commercial or financial relationships that could be construed as a potential conflict of interest.
